# Myocardial infarction in an adult with cystic fibrosis and heart and lung transplant

**DOI:** 10.1186/2049-6958-8-37

**Published:** 2013-06-07

**Authors:** James Eaden, Daniel Peckham

**Affiliations:** 1 Leeds Cystic Fibrosis Unit, St James’s University Hospital, Beckett Street, Leeds, West Yorkshire LS9 7TF, UK

**Keywords:** Cardiac transplant vasculopathy, Cystic fibrosis, Heart and lung transplant, Myocardial infarction

## Abstract

We present a case of myocardial infarction in a 19 year old female with cystic fibrosis who had a heart and lung transplant performed at the age of four years old. She presented atypically with a one day history of severe, intermittent, central, sharp chest pain, radiating to her back and down her left arm. A coronary angiogram showed proximal stenosis of the left anterior descending artery and right coronary artery. She was treated with percutaneous coronary intervention, involving drug eluting stents to the left anterior descending artery (LAD) and the right coronary artery (RCA). In this study we discuss the pathophysiology, investigations and treatment of cardiac transplant vasculopathy. Although complete reversal of LAD and RCA stenosis was achieved, routine follow-up with coronary angiography and careful control of cardiac risk factors will be important to identify and reduce future restenosis and adverse cardiac events.

## Background

Heart and lung transplantation in children with cystic fibrosis (CF) and end-stage lung disease are now rarely performed, with bilateral sequential lung transplantation (BSLT) being the preferred procedure [1]. Although rare, myocardial infarction (MI) in the perioperative period following BSLT has been reported in patients with CF [2-4]. However, to our knowledge there are no reports of a MI occurring years after a heart and lung transplantation in a patient with CF. We present a case of MI in a 19 year old female with CF who had a heart and lung transplant performed at the age of four years old.

## Case presentation

A 19 year old female with CF who had a heart and lung transplantation in 1997 presented to the Emergency Department (ED) with a one day history of severe, intermittent, central, sharp chest pain, radiating to her back and down her left arm. This was associated with intermittent paraesthesia, pallor and coldness in her left hand. The chest pain was worse on deep inspiration but was not positional or exacerbated by movement. Over the previous four weeks she had been having a productive cough with a reduced exercise tolerance but no chest pain.

On examination there was no swelling, pallor or erythema of her left arm/hand and no chest wall tenderness. She was well perfused with a regular pulse and a stable blood pressure. No murmur or added heart sound was present and vesicular breath sounds were heard on auscultation. A chest radiograph showed no acute changes and an initial electrocardiogram (ECG) taken in the ED showed a sinus tachycardia with no ST or T wave changes. Initial blood tests were normal apart from an elevated white cell count (13.64 × 10^9^/L), creatinine (138 μmol/L) and urea (12.8 μmol/L).

The patient was admitted from the ED to the CF ward where she had a repeat ECG and a troponin I blood test after being assessed by a junior doctor. The repeat ECG showed sinus tachycardia with T wave inversion in leads I, aVL, V2-V4, but no ST changes or Q waves. The troponin I level (taken approximately 30 hours after the onset of chest pain) was 10,801. A computed tomography pulmonary angiogram (CTPA) the following day showed no pulmonary embolus (PE) and no vascular abnormalities. A coronary multi-slice computed tomography (MSCT) was not performed as it is not used as an investigation of acute MI in inpatients at St James’s University Hospital.

**Figure 1:**
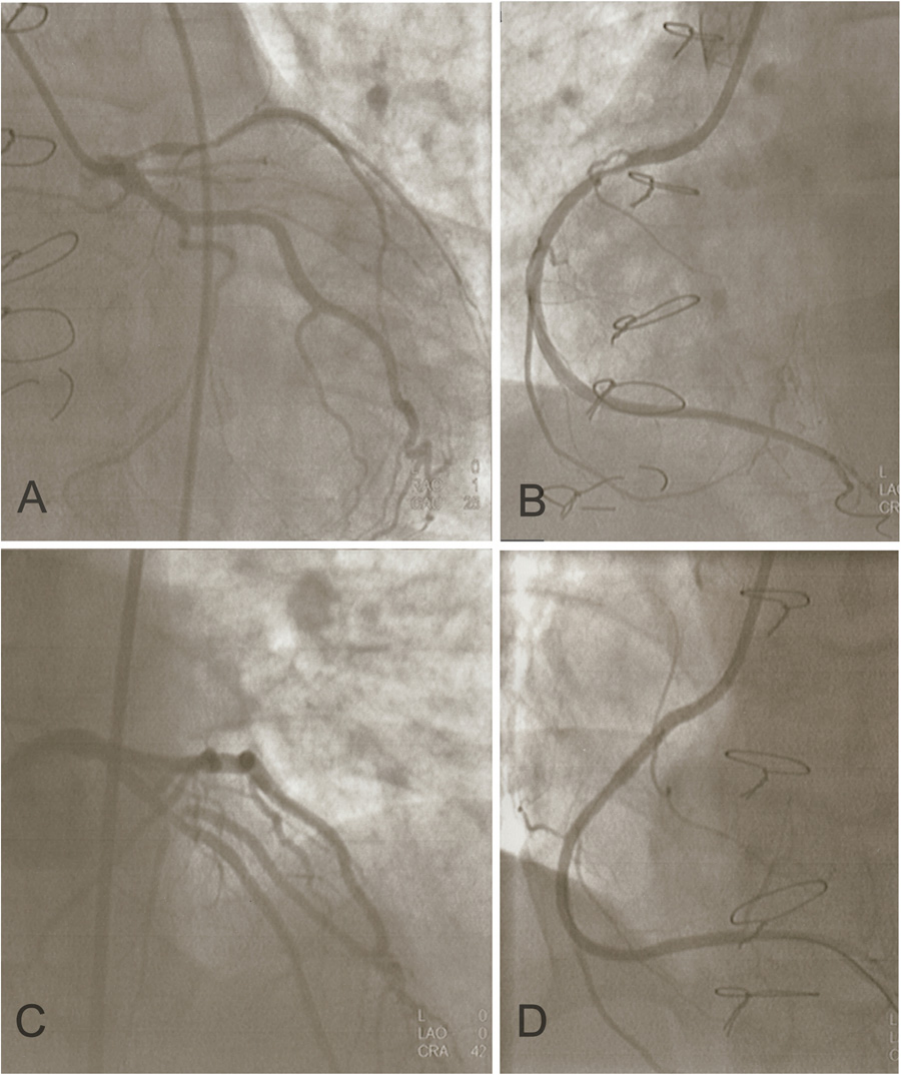
**Angiogram images.** 95% proximal stenosis of LAD (**A**), 80-95% proximal stenosis of RCA (**B**), post-stenting of LAD (**C**), post-stenting of RCA (**D**).

Later that day the patient was reviewed by a cardiology registrar who performed a bedside echocardiogram. This demonstrated moderate left ventricular impairment due to extensive apical akinesis and anterior/antero-septal hypokinesis with an ejection fraction of 42%. No valvular dysfunction was identified. That evening the patient received percutaneous coronary intervention (PCI) which illustrated a 95% proximal stenosis of the left anterior descending artery (LAD), normal left circumflex artery (LCx) and 80-95% proximal stenosis of the right coronary artery (RCA) (Figure 1). Drug eluting stents (DES) were inserted into the LAD and RCA and a complete reversal of the stenosis was achieved. The time between the onset of chest pain and PCI (pain-to-needle time) was approximately 60 hours. This delay in treatment was due to the atypical nature of the chest pain as well as late presentation to the ED. The patient was started on aspirin, bisoprolol, ramipril, lansoprazole and 12 months of ticagrelor and was discharged from hospital five days later.

## Discussion

Cardiac transplant vasculopathy (CTV) is a rapidly progressive form of atherosclerosis involving thickening of the intima, leading to stenosis of coronary artery grafts and subsequent myocardial ischaemia [5]. It is generally a diffuse process that occurs early after transplantation. However, years after transplantation there can be focal atherosclerotic stenosis, diffuse intimal thickening or a combination of both [6]. CTV is a significant complication as it is the leading cause of death in patients with a heart transplant [7].

The development of CTV is thought to be due to a combination of an immune-mediated process and non-immunologic risk factors resulting in endothelial injury and subsequent fibroelastic proliferation of the intima [6]. Risk factors for CTV can be separated into traditional risk factors (hypertension, hyperlipidaemia and diabetes mellitus) and transplant associated risk factors (donor factors, cytomegalovirus (CMV), HLA mismatch and number of rejection episodes) [8]. The patient in this case study did not have diabetes mellitus or hypertension. There was no family history of premature ischaemic heart disease. She was not obese, did not smoke and there was no history of drug abuse. There were no concerns regarding her adherence to medication. She had been taking pravastatin for at least five years and in May 2012 she had a total cholesterol level of 3.5 mmol/L and a triglyceride level of 1.4 mmol/L. There was no evidence of CMV infection, HLA mismatch or rejection and the details of the donor are unknown.

The diagnosis of CTV is particularly challenging as it rarely presents with typical angina, despite 10-30% of patients with heart transplants having partial reinnervation [6]. However, as demonstrated in this case report, CTV can present with atypical chest pain. Many patients with CTV seek medical attention with symptoms of congestive heart failure (e.g. reduced exercise tolerance, dyspnoea on exertion and orthopnoea) and will subsequently have impaired systolic function [6]. Others may present with ventricular arrhythmias or sudden cardiac death [9] and therefore early diagnosis of CTV is important.

In many transplant centres, patients with heart transplants receive annual coronary angiography. However, due to the diffuse nature of CTV, angiography may underestimate the severity of disease or miss it completely [8]. The patient in this case study had a coronary angiogram in 2003 and 2005 which were both normal.

The gold standard investigation of CTV is intravascular ultrasound (IVUS) which has the ability to assess the amount of intimal thickness and identify vessel wall morphology [8]. A study by Mehra et al. [10] involving 74 patients with a heart transplant showed that despite having a normal coronary angiogram, the presence of intimal thickness of more than 0.5 mm on IVUS was associated with more adverse cardiovascular events (death, MI and retransplantation) after approximately four years follow-up. A similar study by Rickenbacher et al. [11] involving 145 patients (one to ten years after heart transplantation) demonstrated a significantly increased four-year overall mortality in patients with a mean intimal thickness of more than 0.3 mm on IVUS compared to those with a mean intimal thickness of 0.3 mm or less. Using serial IVUS Tsutsui et al. [12] found that the majority of intimal thickening occurs within the first year following heart transplantation.

A number of non-invasive radiological modalities (e.g. dobutamine stress echocardiography (DSE), myocardial perfusion imaging (MPI) and multislice computed tomography (CT) coronary angiography) have also been utilized to assist in the diagnosis of CTV, with variable degrees of sensitivity and specificity [8]. A pilot study by Usta et al. [13] involving 10 asymptomatic patients with heart transplants showed that multi-slice spiral CT was equal to coronary angiography in detecting CTV in epimyocardial vessel segments. Multi-slice spiral CT was also more cost-effective with fewer complications when compared with coronary angiography, making it a useful alternative. A study by Spes et al. [14] comparing DSE with coronary angiography and IVUS in 109 patients with heart transplants found that DSE had a sensitivity of 72%. The authors suggested that DSE has a similar prognostic value as IVUS and coronary angiography and that a normal DSE can justify postponing more invasive investigations [14].

The most effective treatment of CTV is prevention and modification of risk factors using antihypertensive agents (e.g. ACE inhibitors and calcium channel blockers), statins (e.g. pravastatin) and strict diabetic control. Coronary artery bypass grafting is rarely an option in CTV due to the diffuse nature of the disease process and the limited availability of donor hearts makes re-transplantation unlikely. In patients with heart transplants presenting with chest pain (atypical or angina) or features of congestive heart failure, if the ECG or echocardiogram show ischaemic changes or wall motion abnormalities, acute coronary syndrome should be suspected and patients should be treated accordingly with antiplatelet agents and PCI [6].

A systematic review of six small, retrospective, nonrandomised studies comparing the use of bare-metal stents (BMS) with drug-eluting stents (DES) in the treatment of CTV (328 lesions treated with BMS and 287 lesions treated with DES) showed that DES were associated with a lower restenosis rate at 6–12 months, but in only half of the six studies were the differences statistically significant [15]. There was no difference in major adverse cardiac events between DES and BMS, but the studies were underpowered and there was considerable variation among the six studies in terms of length of follow-up, type of DES used and the reason for PCI [15]. Less than 20% of the coronary angiograms were performed for possible acute coronary syndromes or stable angina with the main indication being to screen for CTV [15].

A recent retrospective nonrandomised study involving 34 patients receiving a total of 46 stents (27 DES vs 19 BMS) is the first to suggest a clinical benefit (reduced rates of nonfatal MI and cardiac death) with DES compared to BMS in the treatment of CTV [16]. Randomised controlled trials are required to confirm the superiority of DES over BES in the treatment of CTV especially in improving survival or reducing major adverse cardiac events [17].

Maintenance immunosuppression following heart transplantation usually consists of a triple therapy of corticosteroids (usually prednisolone), a calcineurin inhibitor (CNI) (cyclosporine or tacrolimus) and an antiproliferative agent [18]. Azathioprine was the first antiproliferative agent used post heart transplantation; however this has now largely been replaced by mycophenolate mofetil or newer agents such as sirolimus and everolimus. A study involving 273 heart transplant recipients found mycophenolate mofetil to be significantly superior to azathioprine at preventing CTV after five years, when combined with either tacrolimus or cyclosporine [19]. In a randomised, multicentre study involving 634 heart transplant recipients, everolimus was shown to have greater efficacy in reducing the severity and incidence of CTV after one year compared to azathioprine [20]. Sirolimus has also been shown to reduce the progression of CTV either in combination with a CNI [21] or as primary immunosuppression [22,23]. However, the efficacy of everolimus and sirolimus against the progression of CTV in the long-term waits to be seen.

Tacrolimus and cyclosporine have failed to demonstrate prevention of CTV after heart transplantation and there appears to be little difference between these CNIs in terms of long-term development of CTV [24]. However, tacrolimus has been shown to be associated with a lower incidence of hyperlipidaemia and hypertension with no difference in hyperglycaemia, renal function or incidence of infection when compared to cyclosporine [25]. It has also been suggested that tacrolimus monotherapy in heart transplant recipients is safe and efficacious and is not associated with increased high-grade rejection or CTV [26,27].

In terms of CTV prevention, the optimum maintenance immunosupression regime is unclear and a long-term randomised trial is required to determine this. The maintenance immunosupression regime following heart and lung transplantation for the patient in this case consisted of tacrolimus and prednisolone without the use of an antiproliferative agent. There was no change in the maintenance immunosupression regime post MI.

## Conclusion

MI must be considered in young patients with CF and a heart and lung transplant with atypical chest pain. Although complete reversal of LAD and RCA stenosis was achieved in this case with the use of PCI and DES, routine follow-up with coronary angiography and careful control of cardiac risk factors will be important to identify and reduce future restenosis and adverse cardiac events.

## Consent

Written informed consent was obtained from the patient for publication of this case report and any accompanying images.

## Abbreviations

BMS: Bare-metal stents; BSLT: Bilateral sequential lung transplantation; CF: Cystic fibrosis; CFRD: Cystic fibrosis related diabetes; CMV: Cytomegalovirus; CNI: Calcineurin inhibitor; CTV: Cardiac transplant vasculopathy; DES: Drug eluting stents; DSE: Dobutamine stress echocardiography; ECG: Electrocardiogram; ED: Emergency Department; CTPA: Computed tomography pulmonary angiogram; IVUS: Intravascular ultrasound; LAD: Left anterior descending artery; LCx: Left circumflex artery; MI: Myocardial infarction; PCI: Percutaneous coronary intervention; PE: Pulmonary embolus; RCA: Right coronary artery; MPI: Myocardial perfusion imaging; CT: Computed tomography.

## Competing interests

The authors declare that they have no competing interests.

## Authors’ contributions

JE and DP were both involved in the management of the case. JE wrote the case report with assistance from DP. Both authors read and approved the final manuscript.
